# P-1859. A Stewardship Focused Evaluation of Patients Treated with Ceftriaxone for Infections due to Penicillin Susceptible Streptococcus spp. in Outpatient Parenteral Antimicrobial Therapy

**DOI:** 10.1093/ofid/ofaf695.2028

**Published:** 2026-01-11

**Authors:** Jillian M Mack, Nicolás Cortés-Penfield, Richard Hankins, Molly M Miller, Elizabeth Lyden, Melissa LeMaster, Sara Azimi, Scott J Bergman, Trevor C Van Schooneveld, Mark E Rupp, Bryan T Alexander

**Affiliations:** Nebraska Medicine, Omaha, Nebraska; Nebraska Medicine, Omaha, Nebraska; Nebraska Medicine, Omaha, Nebraska; Nebraska Medicine, Omaha, Nebraska; University of Nebraska Medical Center, Omaha, Nebraska; Nebraska Medicine, Omaha, Nebraska; Nebraska Medicine, Omaha, Nebraska; Nebraska Medicine, Omaha, Nebraska; University of Nebraska Medical Center, Omaha, Nebraska; University of Nebraska Medical Center, Omaha, Nebraska; Nebraska Medicine, Omaha, Nebraska

## Abstract

**Background:**

In the outpatient parenteral antimicrobial therapy (OPAT) setting there are limited data quantifying the tradeoffs of using broader spectrum, but more convenient, antimicrobial regimens. This is particularly true with respect to adverse drug events, including *Clostridioides difficile* infection (CDI) and subsequent multidrug resistant (MDR) organism isolation.

**Table 1.**
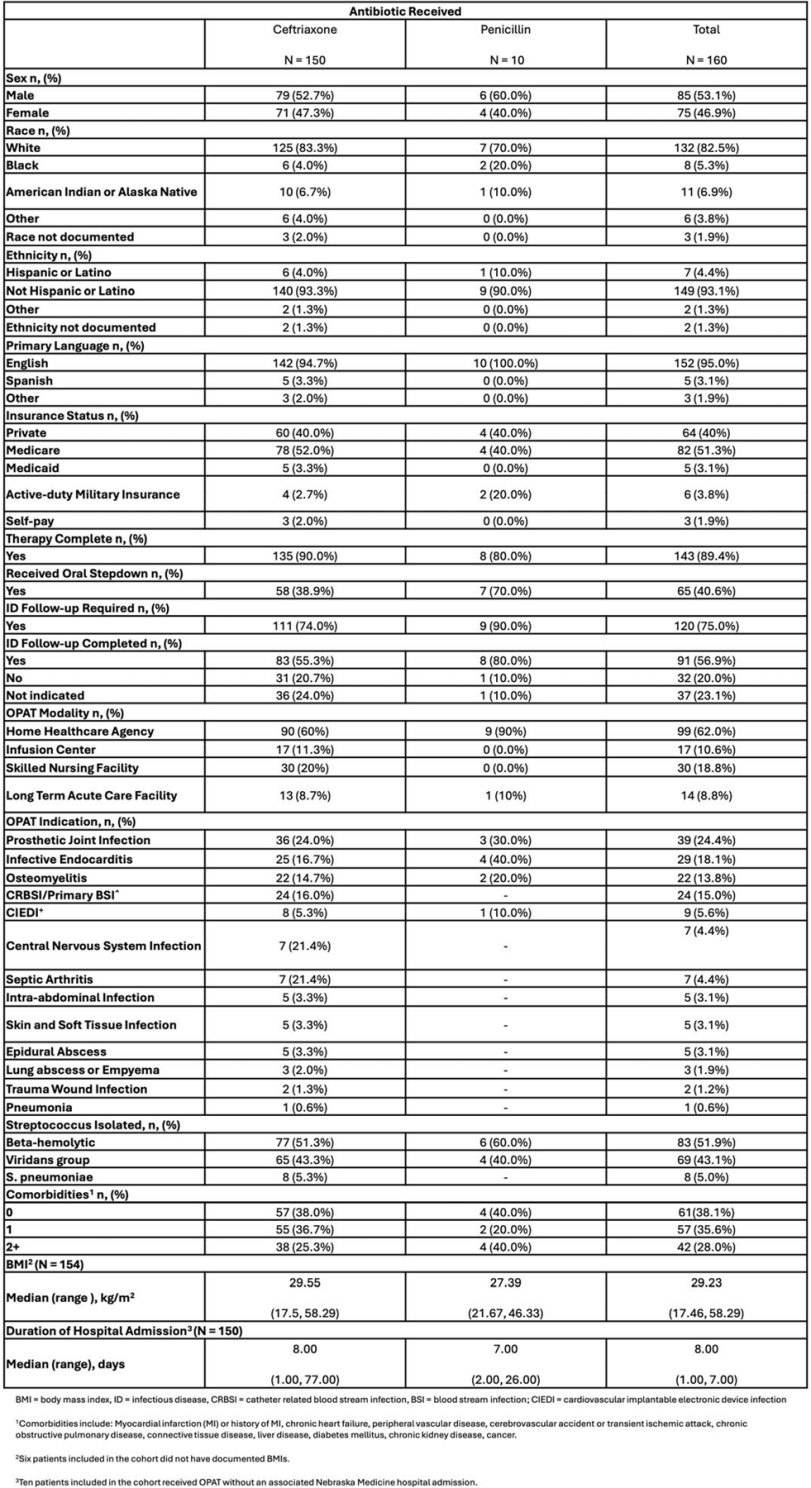
Patient Cohort Characteristics

**Table 2.**
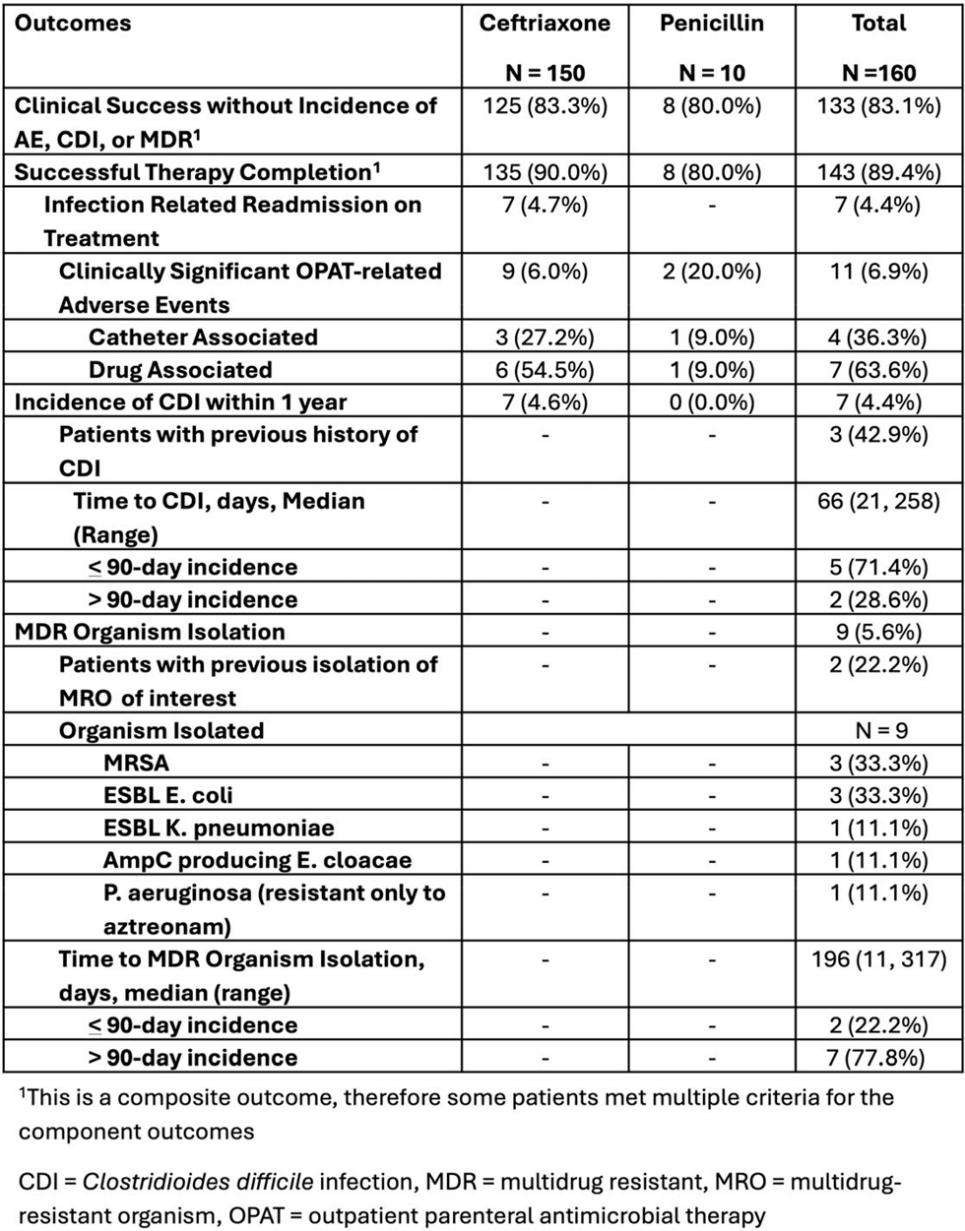
Primary and Secondary Outcomes

**Methods:**

We performed a retrospective cohort analysis of adult patients with confirmed penicillin susceptible streptococcal infection, who received ceftriaxone (CRO) or penicillin through the OPAT service at a large academic medical center. The primary outcome was a composite outcome of clinical success rate without a significant OPAT-related adverse event (AE) or superinfection. The two components of this were successful therapy completion (completion of full planned treatment duration without an infection-related admission or ED visit, no change in therapy due to a drug-related AE, and improved or resolved infection at the end of therapy) and lack of CDI or MDR organism isolation within 12 months of therapy completion. Secondary outcomes included the individual components of the primary outcome.

**Results:**

Patient characteristics reported in Table 1. Outcomes data reported in Table 2. The primary outcome was achieved in 83.1% of patients. Eleven total AEs occurred on therapy; 4 identified as catheter-related and 7 identified as drug-related. There were 1.4 cases of CDI over 1 year per 1000 days of therapy. A total of 9 MDR isolations were identified during the 1-year follow-up period. All CDI and MDR isolations were in CRO treated patients.

**Conclusion:**

Our data are consistent with similar studies demonstrating high rates of clinical efficacy when utilizing CRO to treat highly penicillin-susceptible *Streptococcus* spp. infections. CRO was well tolerated and led to fewer line-related AEs than previously reported with more frequently administered regimens for these infections. Despite CRO being a broader-spectrum agent than necessary for highly penicillin-susceptible *Streptococcus* spp. infections, low adverse event rates, including long-term CDI and MDR isolation, make it an attractive choice for use in the OPAT context.

**Disclosures:**

Trevor C. Van Schooneveld, MD, FSHEA, FIDSA, BioMerieux: Advisor/Consultant|BioMerieux: Grant/Research Support Mark E. Rupp, MD, Armata: Advisor/Consultant|Citius Pharmaceuticals, Inc.: Advisor/Consultant|Magnolia: Grant/Research Support|Teleflex: Advisor/Consultant Bryan T. Alexander, PharmD, BCIDP, AAHIVP, Astellas Pharma: Advisor/Consultant|Merck: Grant/Research Support

